# The effect of resistance training interventions on fundamental movement skills in youth: a meta-analysis

**DOI:** 10.1186/s40798-019-0188-x

**Published:** 2019-05-17

**Authors:** Helen Collins, Josephine N. Booth, Audrey Duncan, Samantha Fawkner

**Affiliations:** 1 Institute of Sport and Exercise, University of Dundee, Old Hawkhill, Dundee, DD14HN UK; 2 Moray House School of Education and Sport, University of Edinburgh, Edinburgh, UK; 3 Physical Activity and Health Research Centre, University of Edinburgh, Edinburgh, UK

**Keywords:** Resistance-training, Children, Adolescents, Movement skills, Strength

## Abstract

**Background:**

Fundamental movement skills (FMS) are strongly related to physical activity (PA) in childhood and beyond. To develop FMS, resistance training (RT) may be a favourable intervention strategy. The purpose of this meta-analysis was to systematically examine the effect of RT interventions on FMS in youth.

**Methods:**

Meta-analysis followed the PRISMA guidelines (Prospero registration number CRD42016038365). Electronic literature databases were searched from the year of their inception up to and including June 2017. The search strategy aimed to return studies that included product and process-oriented measures as a means of assessing FMS. Studies from English language peer-reviewed published articles that examined the effect of RT on indicators of FMS in youth, with participants of school age (5–18 years) were included.

**Results:**

Thirty-three data sets were included exploring five outcomes related to FMS. Studies included only reported product-oriented outcomes. Significant intervention effects were identified for: sprint (Hedges’ *g* = 0.292, 95% CI 0.017 to 0.567, *P* = 0.038), squat jump (Hedges’ *g* = 0.730, 95% CI 0.374 to 1.085, *P* = < 0.001), standing long jump (Hedges’ *g* = 0.298, 95% CI 0.096 to 0.499, *P* = 0.004), throw (Hedges’ *g* = 0.405, 95% CI 0.094 to 0.717, *P* = 0.011) and vertical jump (Hedges’ *g* = 0.407, 95% CI 0.251 to 0.564, *P* = < 0.001). There was variable quality of studies, with 33.3% being classified as ‘strong’.

**Conclusion:**

RT has a positive impact on indicators of FMS in youth but more high-quality studies should be conducted to further investigate the role RT may play in the development of FMS. Additionally, to more comprehensively evaluate the impact of RT on FMS, there is a need for FMS assessments that measure both process- and product-oriented outcomes.

## Key points

- Physical activity guidelines and position statements emphasise the importance of ‘activity to strengthen muscle and bone’ and research suggests that resistance training might have an impact on fundamental movement skills in youth.
- This meta-analysis found that resistance training has a positive effect on, sprint, squat jump, standing long jump, throw and vertical jump.
- Further research is required to investigate the role resistance training may play in the development of FMS and there should be a focus on both process and product-oriented outcomes.

## Background

The positive effects of physical activity on the health and well-being of youth are well established. Appropriate levels of physical activity contribute to the development of healthy musculoskeletal tissues (i.e. bones, muscles and joints), a healthy cardiovascular system (i.e. heart and lungs) and neuromuscular awareness (i.e. coordination and movement control) [1]. They also facilitate maintenance of a healthy body weight, provide various psychological benefits and importantly reduce the risk of several diseases [1].

The current guidelines for children aged 5–18 recommend at least 60 min of daily moderate to vigorous physical activity (MVPA), and minimising the time spent sitting, each day [1, 2]. They also recommend an activity that strengthens muscle and bone, at least 3 days a week [1, 2]. However, despite the guidelines, globally less than 50% of young people are meeting the current physical activity guidelines and physical activity levels demonstrate a decline with age; 25% of 11-year-olds meet the recommendations, compared to just 16% of 15-year-olds [3], which indicates that as children advance to adolescence, physical inactivity (not meeting the recommended 60 min of daily MVPA [1]) becomes ubiquitous.

In offering an explanation for why there may be a decline in physical activity levels, it has been suggested that those who do not enhance their muscular strength and fundamental movement skills (FMS) early in life may not develop in a way that would allow them to participate in a variety of activities and sports with confidence in later life [4]. There is an ambiguity in terminology within the literature used to describe movement skill [5] and therefore based on recommendations [5], FMS can be defined as “an organised series of basic movements that involve a combination of movement patterns of two or more body segments” [6]. FMS are commonly categorised as locomotor (e.g. running, jumping, hopping), stability (e.g. balancing, twisting) and object control (throwing, catching, kicking) [7] which could be described as ‘building blocks’ of more complex movements. In addition, it is important to note that when assessing FMS, product-oriented assessments (such as jump height) and process-oriented assessments (such as movement skills batteries) have been reported in the literature [5]. When assessing FMS as a combination of skills, it is common to see batteries of assessments that are based on the process and quality of movement [8] as well as product-oriented outcomes [9]. The development of process and product outcomes is not necessarily synonymous, and there can be a time lag for an improved process to become autonomous and subsequently translate to the development of the product of the skill [9]. Thus, ideally it is recommended that the assessment of FMS should include both process- and product-oriented measure and therefore provide more of a comprehensive measurement of FMS competence [10].

FMS have been shown to be strongly related to physical activity in childhood and into adulthood. Jaakkola et al., indicated that from early to late adolescence, FMS was a strong predictor of physical activity levels [11]. Supporting this, a systematic review in 2010 [7] reviewed 21 studies which examined FMS competency in children and adolescents and found strong evidence for a positive association with physical activity. Whilst unable to demonstrate causation, these studies suggest that movement competency could play a role in explaining physical activity levels across childhood through to adolescence and adulthood, and that interventions aiming to increase physical activity should target improvements in FMS.

To develop FMS, resistance training may be a favourable intervention strategy. Key organisations (National Strength and Conditioning Association (NSCA), United Kingdom Strength and Conditioning Association (UKSCA),, and The British Association of Sport and Exercises Sciences (BASES)) have developed position statements emphasising why youth should be engaged in resistance training and a key benefit identified in these position statements is the positive effect of resistance training on FMS [12–14]. It has been identified that muscular strength is an essential component of motor skill development [15] and both functional (e.g. changes in motor unit coordination) and structural (e.g. muscular hypertrophy) adaptations as a result of resistance training might bring about changes in motor competency [16] which therefore may be linked to the development of FMS. Despite this, the evidence to support the role of resistance training in developing FMS in youth is not well established.

While not specifically focused on FMS, Harries et al. [17] conducted a systematic review and meta-analysis in 2012 which provided evidence for the role of resistance training in improving indicators of FMS via the assessment of product-oriented assessments (vertical jump and sprint). Pooling data from 14 studies, a significant effect of resistance training on vertical jump (mean difference 3.09(95% CI 1.65, 4.51), *Z* = 4.23 (*P* < 0.0001)) was found, suggesting that resistance training has a positive impact on jumping as a movement skill. However, some of the interventions included in this review also involved plyometric training, which is specifically designed to improve power [18] and the focus of the review was on performance gains, i.e. product-oriented measures rather than on the quality of the movement per se. Additionally, all of the participants in this review were athletic adolescents, so this does not provide information regarding younger children or the possible role of resistance training in those who do not take part in organised sport.

There have also been two meta-analyses to date that have investigated the impact of resistance training specifically on athletic performance, in the form of motor skills (running, jumping and throwing) in both children and adolescents [16, 19]. These reviews also had a focus on product-oriented measures and included studies that implemented plyometric training. Behringer et al. [16] conducted a large meta-analysis of 34 studies with the mean age of all analysed participants being 13.2 ± 3.12 years. A combined mean effect of resistance training was reported for running, jumping and throwing (Hedges’ *g* = 0.52, 95% CI 0.33–0.71). Effect sizes for each of the individual aforementioned skill types were Hedges’ *g* = 0.54, 95% CI 0.34–0.74, Hedges’ *g* = 0.53, 95% CI 0.23–0.83, and Hedges’ *g* = 0.99, 95% CI 0.19–1.79 respectively. It was also shown that younger children and non-athletes demonstrated higher gains. This meta-analysis therefore provides evidence that a resistance training intervention could have a positive effect on FMS in youth and importantly, non-athletic participants. Supporting these findings, Lesinski et al. [19] conducted a meta-analysis that included 43 studies with a participant age range of 6–18 years. However, in this study all of the participants were athletes. The analyses using the weighted standardardised mean difference revealed moderate effects of resistance training on vertical jump (SMD_wm_ = 0.80; *I*^2^ = 67%; *χ*^2^ = 137.47; df = 46; *P* < 0.001), and small effects on linear sprint (SMD_wm_ = 0.58; *I*^2^ = 41%; *χ*^2^ = 55.74; df = 33; *P* < 0.01). The authors concluded that resistance training was effective for improving proxies of physical performance in youth athletes, but with the caveat that most studies were at high risk of bias [19].

In the context of health, there is currently limited review-level evidence to support the isolated impact of resistance training interventions on FMS in non-athletic youth and in particularly limited resistance training studies including process-oriented measures. Therefore, the purpose of this review was to systematically examine the effect of resistance training interventions on fundamental movement skills in youth.

## Methods

### Search strategy

The search strategy and inclusion criteria were specified and documented in advance on PROSPERO (number CRD42016038365). The conduct and reporting of this review adhered to the guidelines outlined in the PRISMA statement [20].

Electronic literature databases were searched from the year of their inception up to and including June 2017. These were PubMed, MEDLINE, ERIC, PsycINFO, Embase, SPORTDiscus and Scopus. Relevant references from published literature were followed up and included where they met the inclusion criteria and literature not identified in the electronic searches was sourced. ResearchGate was used to identify research papers written by key researchers in the field. Additionally, these researchers were contacted regarding any literature not yet published, and the authors of this review searched their personal libraries.

The search terms were related to fundamental movement skills, youths and resistance training (see Table 1). The Boolean operator “AND” was used between search categories and the operator “OR” was used within categories. The search strategy was adapted for each database, and searches were logged.

**Table 1:** Systematic review search categories and terms

Youth^a^, young, child^a^, teen^a^, adol^a^, pube^a^, boys, girls	Resistance training, resistance program^a^, resistance intervention, resistance exercise, weight training, strength and conditioning	Movement, motor, skill, locomotor, physical-performance, athletic-performance, object-control, stability, hop, jump, run, sprint, throw, balance, kick

^a^Search term truncated

Titles of potentially relevant articles were retrieved using the search strategy, duplicates were removed, and then titles and abstracts were screened by HC. Ten percent (*n* = 552) of the titles and abstracts were screened by SF. The inter-rater reliability for the two reviewers was found to be kappa = 0.819 suggesting a strong level of agreement [21]. Full-text copies were obtained for potentially eligible articles and assessed by HC and SF. During the review of full-text articles, a majority decision was taken in consultation with the other reviewers when disagreements regarding inclusion/exclusion occurred.

### Inclusion/exclusion criteria

Studies with participants of school age, between 5 and 18 years were included. No studies were included where the subject group was identified as having a pathological condition or disability which affects movement, such as cerebral palsy or dyspraxia and no studies were included where the subject group was identified as having a behavioural or neuropsychological condition such as autism or attention deficit hyperactivity disorder (ADHD). There may be differential adherence, impact and need for different programmes for groups of children with these identified conditions, so they were excluded from the searches. However, an avenue for future work could be to examine these groups but it was out of the scope of this review.

To allow an isolated review of resistance training, all included studies employed resistance training methods but were excluded if they contained plyometric, vibration or neuromuscular training, or training specifically for rehabilitation purposes. Although these modes of training may also be viewed as forms of resistance training, this review aimed to investigate if isolated strength exercises alone had an effect on FMS. There was no restriction on location (e.g. school-based or sports centre) or timing (e.g. during or after school).

Although studies were included that used a control group and also those that did not, for the purpose of this paper, the analysis focused solely on studies that included a control group, and therefore are referred to as controlled trials (CTs).

It is important to note that although the aim was to include both process- and product-oriented measures of FMS,, no studies included process-oriented measures and therefore only product-oriented measures of jump height/force, throw distance/velocity and sprint times were included. There were not sufficient data to include the stability component of FMS in the meta-analysis nor FMS batteries of assessments.

### Data extraction

Data were extracted using an electronic form by HC and included study characteristics (e.g. country, year); participant characteristics (e.g. sample size, age, sex); intervention components (e.g. setting, duration, content); and changes in the outcomes (e.g. change in fundamental movement skills). The outcome data were extracted in the form of mean, standard deviation and sample size. To check reliability, a second reviewer carried out data extraction on 10% of the included studies, and any disagreements were resolved through discussion with all authors.

### Methodological quality and risk of bias assessment

The “Quality Assessment Tool for Quantitative Studies” developed by the Effective Public Health Practice Project in 1998 [22] was used to assess the quality and risk of bias of the included studies. The results of the assessment led to an overall methodological rating of strong, moderate or weak in eight sections: selection bias, study design, confounders, blinding, data, collection methods, withdrawals and dropouts, intervention integrity and analysis. The assessment tool has been found to be valid and reliable [23]. To check reliability, a second reviewer carried out this assessment on 10% of the included studies, and any disagreements were resolved through discussion between the two reviewers. Overall, the data extraction and risk of bias accuracy of one reviewer was deemed to be acceptable.

### Meta-analysis

Random effects meta-analyses were conducted with the Comprehensive Meta-analysis software (version 2.2.064). Hedges’ *g* with randomised effects and 95% CIs were calculated for trials with sufficient data. The effect size calculations compared the effect of the intervention on the intervention group in comparison to the controls. The magnitude of Hedges’ *g* was interpreted using Cohen’s (1988) convention as small (0.2), medium (0.5) and large (0.8) [24]. A significance level of *P* ≤ 0.05 was applied.

Heterogeneity was assessed using the *I*^2^ statistic. For interpretation, *I*^2^ values of 25, 50 and 75% were considered to indicate low, moderate and high heterogeneity, respectively [25]. Publication bias was assessed by calculating Egger bias statistics [26] and Rosenthal’s fail-safe N [27]. Corresponding funnel plots were created.

A moderator analysis was conducted to determine whether the intervention effects on the outcomes differed by sex of participants (males or females), sex of training group (i.e. the training group was designed for either males, females or mixed sex), sport status (specified sports participants or not), age (< 12 or > 12 years, based on primary and secondary school age split), pubertal stage (<Tanner stage 3 or >Tanner stage 3, based on pre-pubertal/pubertal and pubertal/post-pubertal stages), location (school during physical education (PE), school during free time or community), type of control (no resistance training, normal activity, waitlist) and quality of study (weak, moderate or strong). Additional moderator analyses were planned for ethnicity and supervised compared to self-regulated sessions. However, there was insufficient data to allow these analyses. Although data were also extracted for frequency and duration of interventions, a moderator analysis was not conducted on this data due to the inappropriateness of separating their independent and combined impact on training outcomes.

It is important to note that for outcomes where a decrease in score was a positive intervention effect (e.g. sprint) and an increase in score was a positive effect (e.g. jump) this was accounted for in the analyses to ensure an accurate calculation of effect sizes.

## Results

**Fig. 1:**
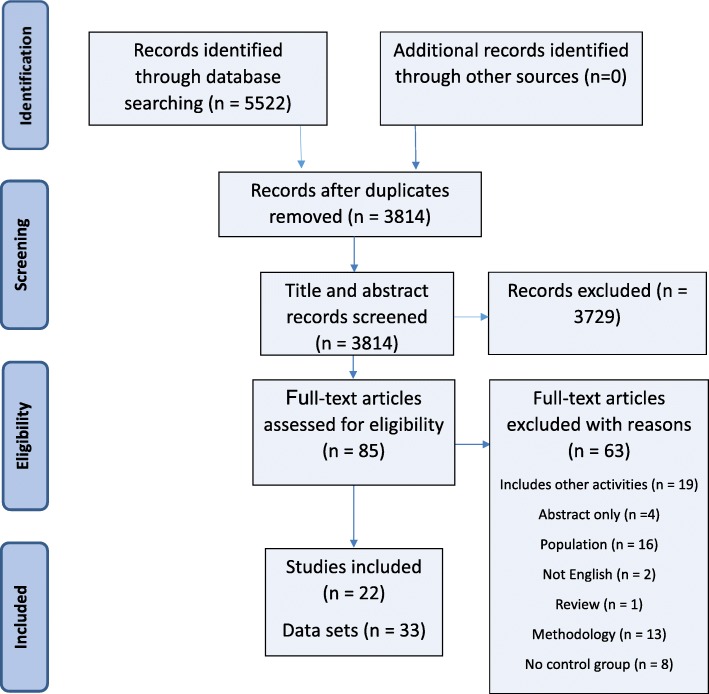
PRISMA flow diagram of systematic search and included studies

Out of an initial 5522 studies identified through database searches, 85 studies met the inclusion criteria (Fig. 1). Following assessment of the full text, 63 studies were excluded from the meta-analysis as they did not meet the inclusion criteria. Twenty two studies and 33 data sets were included in the meta-analysis (some studies had more than one intervention group).

### Study characteristics

Studies were conducted in 11 different countries (Canada, USA, Tunisia, Greece, Spain, Switzerland, Germany, UK, Brazil, Norway and Portugal). There were 542 participants in the experimental groups (sample sizes ranged from 8 to 78 participants) and 401 participants in the control groups (sample sizes ranging from 5 to 76 participants).

The age of all participants ranged from 8 to 17 years. Five outcomes related to fundamental movement skills were included in the analysis: sprint, throw, vertical jump, squat jump and standing long jump. The average attendance figures for the studies that reported it was 89%. The study details can be found in Table 2.

**Table 2:** Description of included studies/data sets

Study	Country		Participant details					Intervention details					
		*N*	Age (years ± sd)	Pubertal stage (Tanner)	Sport/not specified	% males	Sex of training group	Location	Weeks/× per week (min per session)	Sets/reps/intensity	Exercises	Outcomes included	Quality score
Alberga et al. (2015) [38]	Canada	EG 78 CG 76	EG 15.9 ± 1.5 CG 15.6 ± 1.3	4–5	Not specified	29	Mixed	Community	26 weeks × 4(60 min)	2–3 sets, 6–15 reps, max for reps	Whole body exercises on resistance machines + some dumbbell exercises and body weight exercises	Vertical jump	2
Channell et al. (2008) a [39]	USA	EG 11 CG 6	EG 15.9 ± 1.2 CG Not stated	Not stated	Sport	100	Males	Not stated	12 weeks × 3	3–5 sets, 3–20 reps, 60–95% 1RM	Day 1: bench press, power clean, push jerk, leg press, incline, push ups, back extensions abs. Day 2: bench press, power clean, push jerk, lunges, decline, push ups, back extensions, abs. Day 3: bench press, power clean, push jerk, attacker, military press, push ups, back extensions, abs.	Vertical jump	3
Channell et al. (2008) b [39]	USA	EG 10 CG 6	EG 15.9 ± 1.2 CG Not stated	Not stated	Sport	100	Males	Not stated	12 weeks × 3	3–5 sets, 3–20 reps, 60–100% 1RM	Day 1: bench press, squat, deadlift, leg press, incline, push ups, back extensions, abs. Day 2: bench press, squat, deadlift, lunges, decline, push ups, back extensions, abs. Day 3: bench press, squat, deadlift, attacker, military press	Vertical jump	3
Chelly et al. (2009) [40]	Tunisia	EG 11 CG 11	EG 17 ± 0.3 CG 17 ± 0.5	5	Sport	100	Males	Community	8 weeks × 2	4 sets, 2–7 reps, 70–90%1RM	Half back squat	Squat jump, vertical jump	3
Christou et al. (2006) [41]	Greece	EG 9 CG 9	EG 13.8 ± 0.4 CG 13.5 ± 0.5	3–5	Sport	100	Males	Community	16 weeks × 2(45 min)	2–3 sets, 8–15 reps, 55–80%1RM	Leg press, bench press, leg extension, pec-deck, leg flexion, overhead press, lat pull-downs, calf raise, sit-ups, and upper-lower back extension	30-m sprint, squat jump, vertical jump	3
Faigenbaum et al. (1993) [42]	USA	EG 14 CG 10	EG 10.8 CG 9.9	1–2	Not specified	68	Mixed	Not stated	8 weeks × 2 (35 min)	3 sets, 10–15 reps, 50–100%1RM	Leg extension, leg curl, overhead press, bicep curl, chest press	Vertical jump, seated medicine ball put	2
Faigenbaum et al. (1996) [43]	USA	EG 15 CG 9	EG 10.8 ± 0.4 CG 10 ± 0.4	1–2	Not specified	73	Mixed	Not stated	8 weeks × 2	2–3 sets, 6 reps, max for reps	Leg extension, leg curl, chest press, overhead press, bicep curl, ab curl, bent knee leg raise	Vertical jump	1
Faigenbaum et al. (2002) a [44]	USA	EG 22 CG 9	EG 10.2 ± 1.4 CG 9.3 ± 1.5	Not stated	Not specified	68	Mixed	Community	8 weeks × 1(60 min)	1 set, 10–15 reps, max for reps	Leg press, leg extension, leg curl, seated chest press, chest crossover, lat pull down, seated row, shoulder press, biceps curl, and triceps extension, ab curl, lower back extension	Vertical jump, standing long jump	1
Faigenbaum et al. (2002) b [44]	USA	EG 20 CG 9	EG 9.7 ± 1.4 CG 9.3 ± 1.5	Not stated	Not specified	55	Mixed	Community	8 weeks × 2(60 min)	1 set, 10–15 reps, max for reps	Leg press, leg extension, leg curl, seated chest press, chest crossover, lat pull down, seated row, shoulder press, biceps curl, and triceps extension, ab curl, lower back extension	Vertical jump, standing long jump	1
Faigenbaum et al. (2005) a [45]	USA	EG 19 CG 12	EG 10.4 ± 1.5 CG 10.9 ± 0.9	Not stated	Not specified	58	Mixed	Community	8 weeks × 2(40 min)	1 set, 15–20 reps, max for reps	Leg press, squat press, chest press, seated row, overhead press, pulldown, seated dip, ab curl, hip extension	Vertical jump, standing long jump	1
Faigenbaum et al. (2005) b [45]	USA	EG 12 CG 12	EG 10.4 ± 1.2 CG 10.9 ± 0.9	Not stated	Not specified	50	Mixed	Community	8 weeks × 2(40 min)	1 set, 6–10 reps, max for reps	Leg press, squat press, chest press, seated row, overhead press, pulldown, seated dip, ab curl, hip extension	Vertical jump, standing long jump	1
Flanagan et al. (2002) a [46]	USA	EG 8 CG 20	EG 8.8 ± 0.5 CG 8.7 ± 0.5	Not stated	Not specified	48	Mixed	Community	10 weeks x 2(40 min)	1–3 sets, 8–15 reps, max for reps	Squat, bench, pull downs, military press, bicep curls, hamstring curls, tricep pressdown, curl ups	Medicine ball put, standing long jump, shuttle run	2
Flanagan et al. (2002) b [46]	USA	EG 22 CG 20	EG 8.6 ± 0.58 CG 8.7 ± 0.5	Not stated	Not specified	48	Mixed	School -PE	10 weeks × 2(40 min)	Varied–body weight.	Sea crawl, crab crawl, turtle walk, inch worm, treadmill (like a crawl)	Medicine ball put, standing long jump, shuttle run	2
Gorostiaga et al. (1999) [32]	Spain	EG 9 CG 9	EG 15.1 ± 0.7 CG 15.1 ± 0.5	5	Sport	100	Males	Not stated	6 weeks × 2(40 min)	4 sets, 3–12 reps, 40–90%1RM	Supine bench press, half squat, knee flexion curl, leg press and pec-deck	Squat jump, vertical jump, throwing velocity	1
Granacher et al. (2011) [47]	Switzerland	EG 14 CG 14	EG 16.7 ± 0.6 CG 16.8 ± 0.7	4–5	Not specified	48	Mixed	School -PE	8 weeks × 2(90 min)	4 sets, 10 reps, 30–40% 1RM	Leg press, leg extension and flexion, calf raise, weight machine for hip abduction/adduction, back squat	Standing long jump, 20-m sprint	2
Hammami et al. (2017) [48]	Tunisia	EG 16 CG 12	EG 16.2 ± 0.6 CG 16.8 ± 0.2	Not stated	Sport	100	Males	Community	8 weeks × 2(45 min)	3–5 sets, 3–8 reps, 70–90%1RM	Back half squat	Vertical jump, squat jump	2
Hetzler et al. (1997) a [49]	Germany	EG 10 CG 10	EG 13.8 ± 0.6 CG 13.9 ± 1.1	3–4	Sport	100	Males	Community	12 weeks × 3	1–3 sets, 10–12 reps, up to 100% 10RM	Supine bench press, wide grip cable pull down, leg extension, leg curl, leg press, bicep curl, tricep extension, shoulder dumbbell routine, wrist curls, reverse wrist curls	Vertical jump	3
Hetzler et al. (1997) b [49]	Germany	EG 10 CG 10	EG 13.2 ± 0.9 CG 13.9 ± 1.1	3–4	Sport	100	Males	Community	12 weeks × 3	1–3 sets, 10–12 reps, up to 100% 10RM	Supine bench press, wide grip cable pull down, leg extension, leg curl, leg press, bicep curl, tricep extension, shoulder dumbbell routine, wrist curls, reverse wrist curls	Vertical jump	3
Lillegaard et al. (1997) a [50]	USA	EG 20 CG 18	EG 11.2 ± 1.1 CG 10.1 ± 1.6	1–2	Not specified	100	Mixed	Not stated	12 weeks × 3(60 min)	3 sets, 10 reps, 10RM	Barbell curl, tricep extension, leg press, leg curl, lat pulldown, bench press	Vertical jump, shuttle run, 30-yard sprint, standing long jump	3
Lillegaard et al. (1997) b [50]	USA	EG 16 CG 10	EG 14 ± 0.98 CG 13.1 ± 1.6	3–5	Not specified	100	Mixed	Not stated	12 weeks × 3(60 min)	3 sets, 10 reps, 10RM	Barbell curl, tricep extension, leg press, leg curl, lat pulldown, bench press	Vertical jump, shuttle run, 30-yard sprint, standing long jump	3
Lillegaard et al. (1997) c [50]	USA	EG 8 CG 6	EG 9.5 ± 1.4 CG 9.6 ± 1.2	1–2	Not specified	0	Mixed	Not stated	12 weeks × 3 (60 min)	3 sets, 10 reps, 10RM	Barbell curl, tricep extension, leg press, leg curl, lat pulldown, bench press	Vertical jump, shuttle run, 30-yard sprint, standing long jump	3
Lillegaard et al. (1997) d [50]	USA	EG 8 CG 5	EG 12.6 ± 1.6 CG 12.6 ± 1.6	3–5	Not specified	0	Mixed	Not stated	12 weeks × 3 (60 min)	3 sets, 10 reps, 10RM	Barbell curl, tricep extension, leg press, leg curl, lat pulldown, bench press	Vertical jump, shuttle run, 30-yard sprint, standing long jump	3
Lloyd et al. (2016) a [51]	UK	EG 10 CG 10	EG 12.6 ± 0.3 CG 12.8 ± 0.2	Not stated	Not specified	100	Males	School -PE	6 weeks × 2(60 min)	3 sets 10 reps, 10RM	Barbell back squat, barbell lunge, dumbbell step up, leg press	20-m sprint, squat jump	3
Lloyd et al. (2016) b [51]	UK	EG 10 CG 10	EG 16.3 ± 0.3 CG 16.2 ± 0.3	Not stated	Not specified	100	Males	School - PE	6 weeks × 2(60 min)	3 sets 10 reps, 10RM	Barbell back squat, barbell lunge, dumbbell step up, leg press	20-sprint, squat jump	3
Moraes et al. (2013) a [52]	Brazil	EG 14 CG 10	EG 15.5 ± 0.9 CG 15.6 ± 0.9	3–4	Not specified	100	Males	Community	12 weeks × 3	3 sets, 10–12 reps, max for reps	Machine bench press, 45° leg press, front lat pull-down, leg extension, military press, seated leg curl, pulley triceps extension, abdominal crunches, arm curl	Vertical jump, standing long jump	1
Moraes et al. (2013) b [52]	Brazil	EG 14 CG 10	EG 15.4 ± 1.1 CG 15.6 ± 0.9	3–4	Not specified	100	Males	Community	12 weeks × 3	3 sets, 3–20 reps, max for reps	Machine bench press, 45° leg press, front lat pull-down, leg extension, military press, seated leg curl, pulley triceps extension, abdominal crunches, arm curl	Vertical jump, standing long jump	1
Negra et al. (2016) [28]	Tunisia	EG 13 CG 11	EG 12.8 ± 0.3 CG12.7 ± 0.2	1–3	Sport	100	Males	Community	12 weeks × 3(90 min)	4 sets, 8–12 reps, 40–60%1RM	Half back squat	Vertical jump, standing long jump, squat jump, 30-m spring, *t* test	1
Saeterbakken et al. (2010) [33]	Norway	EG 14 CG 10	EG 16.6 ± 3.1 CG 16.5 ± 3.9	Not stated	Sport	0	Females	Community	6 weeks × 2(75 min)	4 sets, 406 reps, max for reps	Spine abduction, side lying plank, dynamic crunch, superman, one leg squat, push ups	Throwing velocity	1
Sander et al. (2012) a [53]	Germany	EG 30 CG 25	EG 15 CG 15	Not stated	Sport	100	Males	Community	104 weeks × 2	5 sets, 4–10 reps, max for reps	Parallel front and back squats, bench presses, deadlifts, neck presses and exercises for the trunk muscles as well as the standing row	30-m sprint	3
Sander et al. (2012) b [53]	Germany	EG 18 CG 33	EG 13 CG 13	Not stated	Sport	100	Males	Community	104 weeks × 2	5 sets, 4–10 reps, max for reps	Parallel front and back squats, bench presses, deadlifts, neck presses and exercises for the trunk muscles as well as the standing row	30-m sprint	3
Santos et al. (2012) [54]	Portugal	EG 15 CG 10	EG 14.5 ± 0.6 CG14.2 ± 0.4	3–4	Sport	100	Males	Community	10 weeks × 2	3 sets, 3–12 reps, max for reps	Day 1: squat, press, upright row, lunge, push up, bicep curl, tricep dip, abs. Day 2: squat, deadlift, chest fly, front raise, row, calf raise, tricep extension, abs. Day 3: jump, high pull, press, upright row, squat jump, lying press, broad jump, abs	Squat jump, vertical jump, medicine ball throw	2
Weltman et al. (1986) [55]	USA	EG 16 CG 10	EG 8.2 ± 1.3 CG 8.2 ± 1.3	> 2	Not specified	100	Males	Community	14 weeks × 3(45 min)	1 set, 30 s on, 30 s off	Biceps, triceps, bench press, quads, hamstrings, shoulder press, hip abduction/adduction, butterfly, forearm conditioner	Vertical jump, standing long jump	2
Zouita et al. (2016) [29]	Tunisia	EG 26 CG 26	13–14	2–3	Sport	100	Males	Community	12 weeks × 3(90 min)	15–20 reps, 30–80%1RM	Squat, bench press, push ups, sit ups	30-m sprint, squat jump, vertical jump, *t* test	2

*EG experimental group, *CG* control group, *rep* repetition, *max* maximum, *RM* repetition maximum, *ab* abdominal, *lat* latissimus dorsi

### Synthesis of results

**Fig. 2:**
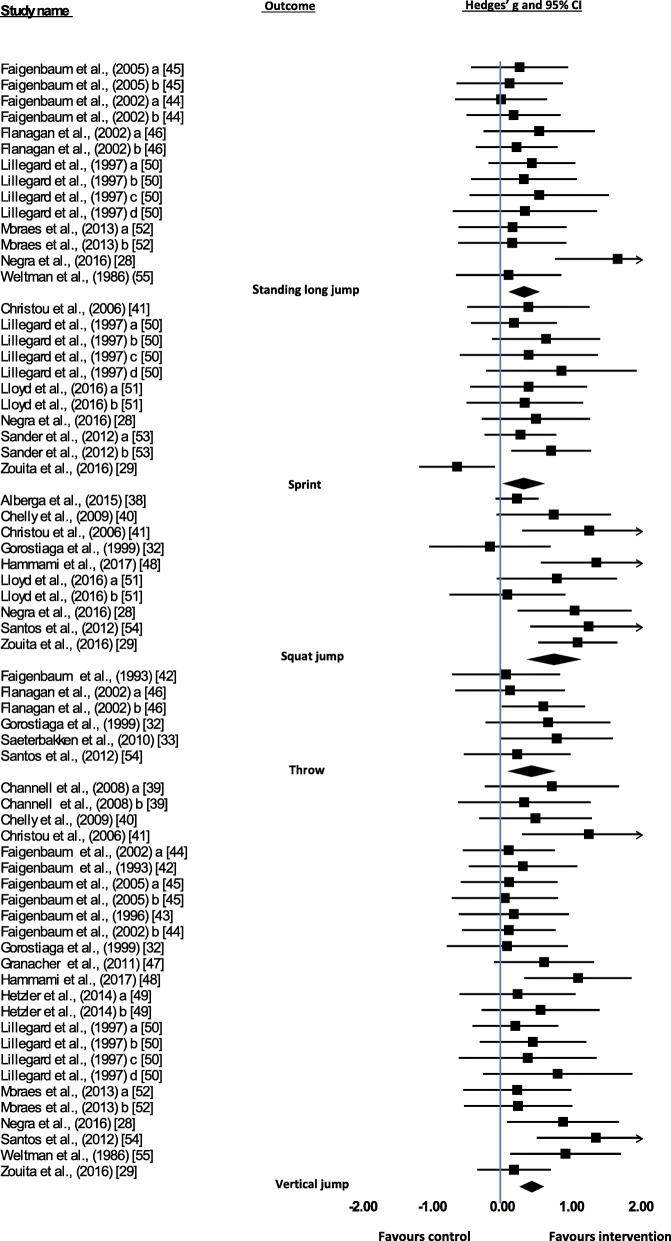
Summary of all fundamental movement skills meta-analyses

For each study, Hedges’ *g* was calculated for each outcome variable to determine an overall intervention effect. Figure 2 illustrates the effect sizes for all of the studies and the overall effect size for each outcome, which ranged from 0.292 to 0.730, indicating a small to medium intervention effect relative to controls. Significant intervention effects were identified for all outcomes. These were sprint (Hedges’ *g* = 0.292, 95% CI 0.017 to 0.567, *P* = 0.038), squat jump (Hedges’ *g* = 0.730, 95% CI 0.374 to 1.085, *P* = 0.000), standing long jump (Hedges’ *g* = 0.298, 95% CI 0.096 to 0.499, *P* = 0.004), throw (Hedges’ *g* = 0.405, 95% CI 0.094 to 0.717, *P* = 0.011) and vertical jump (Hedges’ *g* = 0.407, 95% CI 0.251 to 0.564, *P* = < 0.001). Overall effect sizes for outcomes were in favour of the intervention.

Based on the thresholds [25] moderate heterogeneity was identified for squat jump (*I*^2^ = 59%) and low heterogeneity for all other outcomes (*I*^2^ = 0–35%).

### Publication bias

**Fig. 3:**
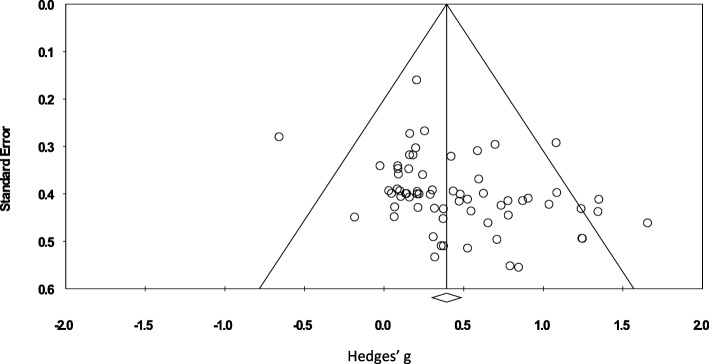
Funnel plot of publication bias

To identify possible publication bias, effect sizes were plotted against standard errors to give funnel plots as illustrated in Fig. 3. This indicated the presence of publication bias which was confirmed by a significant result from Egger’s test [26]. Rosenthal’s fail-safe N [27] found that 1314 additional studies would be needed for the cumulative effect to be non-significant. Therefore, it can be concluded that there is possible publication bias but that it is unlikely to exert a strong influence.

### Quality appraisal

Through the quality assessment process, 33.3% of the studies were classified as ‘strong’, 33.3% were classified as ‘moderate’ and 33.3% were classified as ‘weak’. Investigating the quality of studies as a moderator, the only significant effect was on sprint (Table 3).

**Table 3:** Moderator analysis

Moderator	Outcome	Hedges’*g* (95% CI)	No. of studies	Between group comparison: *Q* (df)
Sex of participants	Squat jump	Males 0.836** (0.497–1.175)	9	7.080 (1)**
		Females 0.207 (− 0.108–0.522)	1	
Sex of training group	Squat jump	Males 0.836** (0.497–1.175)	9	7.080 (1)**
		Mixed 0.207 (− 0.108–0.522)	1	
Sport status	Squat jump	Sport 0.949*** (0.585–1.759)	7	8.891 (1)**
		Not sport 0.251 (− 0.029–0.530)	3	
	Standing long jump	Sport 1.658 *** (0.752–2.564)	1	9.107 (1) **
		Not sport 0.227* (0.020–0.433)	13	
Type of control	Standing long jump	No strength 0.227*(0.020–0.433)	13	9.107 (1)**
		Sport only 1.658**(0.752–2.564)	1	
Quality score	Sprint	1 (strong) = 0.481 (− 0.306–1.268)	1	12.575 (2)**
		2 (moderate) = − 0.657*(−1.207–0.107)	1	
		3 (weak) = 0.418** (0.173–0.663)	9	

*P < 0.05, **P < 0.01, ***P < 0.001

### Moderator analysis

Moderator analysis followed to determine whether the intervention effects on each outcome variable differed by sex of participants, sex of the training group, sport status, age, pubertal stage, type of control group activity and the quality score of the studies. The significant findings are shown in Table 3. There was a selected impact of sex, sport status, type of control and quality on some of the outcomes, most commonly on squat and long jump. These moderators did not have an impact on all outcomes though. Age and pubertal status were not found to statistically moderate the impact of interventions on any of the outcomes examined. However, it should be noted that there were not equal numbers of studies in all comparisons, which may have had an impact on the findings.

## Discussion

### Summary of evidence

The UKSCA [13] and NSCA’s [12] position statements on youth resistance training both suggest that resistance training may have a positive impact on fundamental movement skills. This is the first meta-analysis reported that focusses solely on resistance training, with the aim of examining the impact of resistance training on fundamental movement skills in youth. Analysis indicated that resistance training has a positive impact on a number of fundamental movement skills as assessed by product-oriented measurement outcomes. Statistically significant effect sizes were found for all of the FMS outcomes included a medium effect of resistance training interventions on squat jump and a small effect on all other outcomes (vertical jump, standing long jump, sprint and throw).

It has been identified that muscular strength is an essential component of motor skill development [15] and both functional (e.g., changes in motor unit coordination) and structural (e.g., muscular hypertrophy) adaptations as a result of resistance training might bring about changes in motor competency [16] which therefore may be linked to the development of FMS. In particular, neural adaptations as a result of resistance training include changes in motor unit coordination, firing and recruitment, which are factors that are known to be essential for optimal movement, and likely to play a major role in reported changes, especially in younger children for whom hypertrophy is less likely [16].

Reinforcing this, it has been reported that increases in sprint performance due to resistance training are most likely caused by increases in neuromuscular activation of the trained muscles [28, 29]. Thus, there appears to be strong evidence from this meta-analysis to support the role of resistance training to enhance outcomes commonly associated with FMS in youth, which might be a logical assumption when strength is reported to be an essential component of motor skill competency [15].

The largest effect size in this meta-analysis was for the squat jump. This meta-analysis included only isolated resistance training interventions, suggesting that these effects occur with resistance training in the absence of any form of power training (such as plyometrics) and therefore this may explain the larger effect on a single squat jump which does not involve a plyometric element (counter movement) in comparison to the vertical jump and standing long jump, which do. In support of this, van Hooren et al. identifies that “In the CMJ, the athlete starts from a standing position and initiates a downward movement, which is immediately followed by an upward movement leading to takeoff. In contrast, during the SJ, the athlete descends into a semi-squat position and holds this position for approximately 3 seconds before takeoff.” [30] Therefore, this clarifies the difference between the two assessment outcomes of squat jump and vertical jump.

Plyometrics as a mode of training is included in previously published reviews and scrutiny of the effect sizes across the studies suggests that inclusion of plyometrics leads to greater enhancement of FMS. Behringer et al. [16] reported a medium effect size for jumping; however, this was both vertical jump and standing long jump combined, and it was not specified whether the vertical jump had a counter movement, or whether the analysis included squat jumps, which do not have a plyometric element. Harries et al. [17] reported a positive effect of resistance training on vertical jump performance (mean difference (MD) = 2.09, 95% CI − 0.01 to 4.20, Z = 1.95, *P* = 0.05) and a larger effect for studies that combined plyometric with resistance training (MD = 3.03, 95% CI = 0.83 to 5.24, *Z* = 2.69, *P* = 0.007) or included plyometric training alone (MD 5.47 [1.95, 9.00], Z = 3.04 [*P* = 0.002]). Lesinski et al. [19], who also included plyometrics as a training mode, similarly reported a large, significant effect size for vertical jump (SMD_wm_ = 0.80; *I*^2^ = 67%; *χ*2 = 137.47; df = 46; *P* < 0.001) (although again it is not clear whether this included a counter movement, or whether squat jumps were also included in the analysis). Also, whilst Lesinski et al. [19] did exclude uncontrolled trials, direct comparisons with the present review are not wholly appropriate as several of the studies included in their review involved plyometric training. It is not surprising that plyometric exercise including jumping would result in an improvement in jump performance. However, the results from the current meta-analysis would suggest that the development of strength has a key role to play. This strength development could be associated with the quality and coordination of movement rather than power development alone, which could be more relevant for non-athletic populations by producing better movement and therefore more positive physical activity experiences.

For the current review, it is important to be cautious when drawing conclusions from the jumps data both because of the number of studies (there were 25 vertical jump data sets compared to 10 squat jump and 14 standing long jump data sets) and because of the high heterogeneity across the studies that included the squat jump. The moderator analysis indicates that this may be explained by sex and sport status and is discussed further below.

For the outcome of the sprint, there was a small, significant effect which suggests that adaptations occur that might impact on speed. Supporting this, studies have shown significant correlations between maximal squat strength and sprint performance in youth [18, 31]. Behringer et al. [16] reported an effect size of 0.54 (95% CI 0.34–0.74), which included both shuttle runs and straight sprints and similar results were published by Lesinski et al. [19] (SMD_wm_ = 0.58; *I*^2^ = 41%; *χ*2 = 55.74; df = 33; *P* < 0.01) Taken together, these findings imply that resistance training has a positive impact on sprint performance.

There was a small but significant effect size for throw outcomes; however, Behringer et al. [16] reported a large effect size for throwing 0.99 (95% CI: 0.19–1.79). In the present review, it is important to note that out of the 6 data sets, two included a handball throw [32, 33]. This task is sport specific and therefore the specific technique required to play the sport may have influenced the results.

It should be noted that both the reviews from Behringer et al. [16] and Harries et al. [17] combined controlled trials and uncontrolled trials for the analyses, which has implications for comparing results to the present review. For uncontrolled trials, it is difficult to ascertain if any intervention effects are due to the normal process of growth and maturation. Equally, for the studies that include participants taking part in performance sport, the effect of normal training cannot be controlled for; to investigate intervention effects in youth populations it is critical to include a control group to ensure appropriate interpretation of results.

Given the lack of studies that investigated the role of isolated resistance training in improving FMS using process-oriented assessment batteries, the current review instead examined individual product oriented FMS outcomes. Nevertheless, we have demonstrated that resistance training has a significant effect on all assessed outcomes, which suggests a positive effect on overall movement. This has positive implications for creating strategies to develop FMS and ultimately encourage a healthier and more active lifestyle.

### Moderator analysis

To investigate the findings further, a moderator analysis was completed on all outcomes to identify if any effects could be explained by specific moderator variables. It was found that the sex of participants was a moderator for squat jump, sex of training group was a moderator for squat jump, sport status was a moderator for squat jump and standing long jump, the type of control group was a moderator for the standing long jump, and additionally quality score was a moderator for sprint (see Table 2).

#### Sex of participants and sex of training group

The outcome of squat jump displayed high heterogeneity. The sex of participants (males or females) and sex of training group (i.e. the training group was designed for either males, females or mixed sex) may explain this variance, with more of an effect on males and the male training groups.

In adolescents, it has been reported that during puberty, sex differences in muscular strength occur with boys demonstrating accelerated gains [34]. However, it has been suggested that there is no clear evidence of any difference in strength between pre-pubescent girls and boys [35]. As this meta-analysis included both children and adolescents, it is difficult to make conclusions based on this data. Additionally, for squat jump there was only one study that included females and nine that included males.

#### Sport status

For squat jump, and standing long jump distance, there was more of an effect of resistance training on those involved in sport compared to those who were not identified as being involved in a specific sport (e.g. identified as ‘school children’). Recent research has found an association between FMS and participation in organised sports [36]. Those study participants who take part in sport may therefore already have well-developed FMS at baseline, greater competency with the resistance training, and therefore would be more susceptible to further gains. Those who do not participate in sport might not display as much competency in their movement at baseline and therefore it could take longer to make observable improvements. However, it is important to note that the ‘not sport’ group may have included children who take part in sport; it was just not reported in the study as a ‘sport’ group (e.g. a football team).

#### Age and pubertal stage

There was no moderator effect of age or pubertal stage on any of the outcomes and although Behringer et al. [16] proposed that younger children may experience a greater effect of resistance training due to the degree of neuromuscular adaptation that occurs, Lesinski et al. [19] reported no difference in the effect between pubertal stages or for chronological age. These previous reviews have examined effects in athlete groups, so taken together, it appears that gains in FMS are likely, irrespective of age and maturity status. Morgan et al. [37] identified that some children (particularly older) may experience a ‘ceiling effect’ with some FMS measures. However, ceiling effects are less likely to occur with product assessments because there is always the possibility of performing better when the scoring is related to speed, distance or accuracy [37].

#### Type of control and quality score

There was a large imbalance of studies for type of control group, with 13 studies being ‘no strength control’ versus only 1 study being ‘sport only’. For the quality score, there were nine studies that were ‘weak’ versus only one study being ‘moderate’ and only one study being ‘strong’ and therefore it is not possible to make conclusions based on this data. In particular for the quality score, with only one study being strong, this has implications for interpreting the results as well as suggesting that more quality studies should be undertaken to investigate this topic further.

### Strengths and limitations

There were a number of strengths of this review. There should be strong confidence in the main findings given the rigorous review process. Strict inclusion/exclusion criteria resulted in an analysis of 33 data sets that examined the effects of resistance training on FMS in 542 youths from 11 countries. Additionally, it is the first review to have included resistance training only interventions, rather than include interventions that include plyometric training, which may be more relevant for a sporting population who may be aiming to improve performance.

This review builds on previous reviews, but with the inclusion of non-sporting populations. The context of this review was that resistance training might be a worthwhile intervention to help improve FMS in inactive youth; thus the inclusion of non-sporting participants was important. Although the meta-analysis conducted by Behringer et al. [16] also included non-athletes, 7 years later, an update to build on the data is beneficial.

There was high compliance reported in the included studies. For the studies who reported it, compliance was 89%. As well as a strength of the current review, high compliance adds substance to the potential for resistance training as a viable mode of intervention to improve FMS.

There are also limitations apparent that need to be considered when interpreting the results. There was large variability within the study interventions with regards to participant numbers (ranging from 5 to 78 participants), frequency, duration and programme content. The frequency ranged from 1 to 3 times a week and duration ranged from 6 to 104 weeks. Programmes also involved a mixture of sets and reps with a range of intensities. The forest plot (Fig. 3) also signifies large variation in the individual studies’ results. There was also an indication of the presence of publication bias which should be considered when interpreting the results.

A limitation of the moderator analysis was that not all of the studies reported data to enable a thorough investigation, and there were not equal numbers of studies in all comparisons, so limited conclusions can be made based on this additional level of analysis. Evaluating the quality of the papers included, there was found to be a mixture of quality of studies, with only 33.3% of the studies classified as strong.

Finally, all of the studies included used product-oriented, rather than process-oriented, outcomes. Therefore this meta-analysis does not inform us about how the movements are performed. This supports previous research that has concluded that the use of process and product assessments should be used to comprehensively capture levels of movement competency in human movement [9, 10].

## Conclusions

We are able to conclude that resistance training is likely to have a positive effect on FMS in untrained youth. Overall, significant intervention effects were identified for all outcomes. These were sprint (Hedges’ *g* = 0.292, 95% CI 0.017 to 0.567, *P* = 0.038), squat jump (Hedges’ *g* = 0.730, 95% CI 0.374 to 1.085, *P* = < 0.001), standing long jump (Hedges’ *g* = 0.298, 95% CI 0.096 to 0.499, *P* = 0.004), throw (Hedges’ *g* = 0.405, 95% CI 0.094 to 0.717, *P* = 0.011) and vertical jump (Hedges’ *g* = 0.407, 95% CI 0.251 to 0.564, *P* = < 0.001).

This review provided an overview of the current evidence and therefore has given insight into the potential benefits of such interventions. Although we are able to conclude that resistance training interventions have a positive impact on indicators of FMS, this reflects only a small body of published work.

Based on the findings of this review, and in support of the conclusions of previous reviews, future studies should be designed as randomised controlled trials with large samples and include a treatment group with an isolated resistance training intervention. There should be careful consideration given to appropriate intervention content and assessment methods. Additionally, in the context of increasing physical activity levels, there is a need for assessments that measure both process and product-oriented outcomes.

With resistance training interventions offering potential benefits for youth with regard to FMS, it is imperative that robust and quality studies should be conducted to further investigate the role resistance training may play in the development of FMS. This could ultimately help inform the development of interventions aiming to increase youth physical activity and improve the health of individuals not only during childhood but as they progress through life.

## Abbreviations

BASES: British Association of Sport and Exercise Sciences
NSCA: National Strength and Conditioning Association
PA: Physical activity
RT: Resistance training
UKSCA: United Kingdom Strength and Conditioning Association
